# Rate of Decompensation of Normoxic Emergency Department Patients with SARS-CoV-2

**DOI:** 10.5811/westjem.2020.12.49206

**Published:** 2021-04-02

**Authors:** Kraftin E. Schreyer, Derek L. Isenberg, Wayne A. Satz, Nicole V. Lucas, Jennifer Rosenbaum, Gregory Zandrow, Nina T. Gentile

**Affiliations:** Lewis Katz School of Medicine at Temple University, Department of Emergency Medicine, Philadelphia, Pennsylvania

## Abstract

**Introduction:**

As of October 30, 2020, severe acute respiratory syndrome coronavirus 2 (SARS-CoV-2) has infected over 44 million people worldwide and killed over 1.1 million people. In the emergency department (ED), patients who need supplemental oxygen or respiratory support are admitted to the hospital, but the course of normoxic patients with SARS-CoV-2 infection is unknown. In our health system, the policy during the coronavirus 2019 (COVID-19) pandemic was to admit all patients with abnormal chest imaging (CXR) regardless of their oxygen level. We also admitted febrile patients with respiratory complaints who resided in congregate living. We describe the rate of decompensation among patients admitted with suspected SARS-CoV-2 infection but who were not hypoxemic in the ED.

**Methods:**

This is a retrospective observational study of patients admitted to our health system between March 1–May 5, 2020 with suspected SARS-CoV-2 infection. We queried our registry to find patients who were admitted to the hospital but had no recorded oxygen saturation of <92% in the ED and received no supplemental oxygen prior to admission. Our primary outcome was decompensation at 72 hours, defined by the need for respiratory support (oxygen, high-flow nasal cannula, non-invasive ventilation, or intubation).

**Results:**

A total of 840 patients met our inclusion criteria. Of those patients, 376 (45%) tested positive for SARS-CoV-2. Sixty patients (7.1%) with suspected COVID-19 required respiratory support at 72 hours including 27 (3%) of confirmed SARS-CoV-2 positive patients. Among the 376 patients who tested positive for SARS-CoV-2, 54 patients (14%) had normal CXR in the ED. One-third of patients with normal CXRs decompensated at 72 hours. Seven SARS-CoV-2 positive patients in our cohort died during their hospitalization, of whom five had normal CXRs on admission.

**Conclusion:**

Sixty (7.1%) of suspected COVID-19 patients hospitalized at 72 hours required respiratory support despite being normoxic in the ED. Further research should look to identify the normoxic SARS-CoV-2 patients at risk for decompensation.

## INTRODUCTION

The emergence of severe acute respiratory syndrome-coronavirus-2 (SARS-CoV-2) was first reported in Wuhan, China, in December 2019.^1^ The first case of the disease caused by SARS-CoV-2, named coronavirus 2019 (COVID-19), in the United States was reported in the state of Washington on January 21, 2020.^2^ As of March 29, 2021, the SARS-CoV-2 virus has infected over 126.8 million people worldwide and killed over 2.7 million people.^3^ In the United States, SARS-CoV-2 has infected over 29.9 million people and killed over 543,800 people.

SARS-CoV-2 causes a range of symptoms from mild respiratory illness and gastrointestinal illness to respiratory failure.^4^,^5^ SARS-CoV-2 often causes a biphasic syndrome where respiratory symptoms predominate early during the viremic phase, a quiescent phase, and then subsequent severe inflammatory stage.^6^ In one study from China, the infectious symptoms indicative of the initial phase lasted between 7–10 days. By 10 days, half of all patients had defervesced; most had cleared their fever by week two.

In our health system, the policy during the COVID-19 pandemic was to admit all patients with abnormal imaging and suspected SARS-CoV-2 infection regardless of their oxygen level. We also admitted febrile patients with respiratory complaints who were homeless or resided in congregate living (eg, homeless shelters or recovery houses). This gives our health system a unique perspective as many other hospitals screened away patents who were normoxic without further testing or treatment. Our goal in this study was to describe the clinical course of patients with suspected COVID-19 along with the rate of decompensation of patients with suspected COVID-19 who were not hypoxemic in the ED.

## METHODS

This was a retrospective observational study of all patients admitted to the Temple University Health System between March 1–May 5, 2020. The Temple University Health System (TUHS) is a three-hospital system located in Philadelphia, Pennsylvania. Temple University Hospital (TUH) is a tertiary care hospital located in Philadelphia and is the referral center for the health system; TUH houses a 52-bed emergency department (ED) that is staffed with board-certified emergency physicians. It is the main site for the three-year emergency medicine (EM) residency. Episcopal Hospital is a 29-bed urban community ED, also staffed by board-certified emergency physicians, and is a community site for the EM residency. Episcopal Hospital houses a 19-bed observation unit, but any patients admitted from the Episcopal Hospital ED who need a higher level of care or consultative services are transferred to TUH. The TUH Jeanes Campus is a 19-bed suburban community ED on the outskirts of Philadelphia. Among all three EDs, there were approximately 193,000 ED visits in 2019.

A standard admission order set was used for all patients admitted with suspicion of COVID-19. The order set included laboratory tests, chest imaging (CXR), an electrocardiogram, a SARS-CoV-2 nasopharyngeal polymerase chain reaction (PCR) swab, and oxygen therapy via nasal cannula. Beginning March 1, 2020, patients admitted to any of the three hospitals in the TUHS were entered into a COVID-19 registry if they had a COVID-19 nasopharyngeal PCR performed or had a diagnosis of viral pneumonia or SARS-CoV-2-related illnesses (*International Classification of Diseases, 10^th^ Revision* [ICD-10] codes B97.29, J22, or Z20.828). We queried the registry to find patients who were admitted to the hospital but had no recorded oxygen saturation of less than 92% in the ED and received no supplemental oxygen before admission to the hospital.

**Population Health Research Capsule**

What do we already know about this issue?

*Some patients with COVID-19 pneumonia will decompensate and require oxygen and ventilatory support despite being normoxic in the emergency department (ED).*

What was the research question?

*What is the rate of decompensation of patients with COVID-19 pneumonia who are normoxic in the ED?*

What was the major finding of the study?

*Among 870 patients with COVID pneumonia who were normoxic in the ED, 7% required oxygen or ventilatory support at 72 hours.*

How does this improve population health?

*A significant number of patients with COVID-19 will decompensate at 72 hours despite normoxia in the ED. Further research needs to identify these at-risk patients.*

We included all patients 18 years and older who had a discharge diagnosis of viral pneumonia or SARS-CoV-2 related illnesses (ICD 10 B97.29, J22, or Z20.828); a nasopharyngeal PCR test for SARS-CoV-2; and a CXR or computed tomography (CT) of the chest performed. Patients were excluded if they had a documented oxygen saturation of less than 92% prior to hospital admission, required oxygen while in the ED, were on home oxygen at baseline, had no SARS-CoV-19 test performed, or had no radiology studies of the chest performed. Patients were also excluded if they were less than 18 years old, a prisoner, or pregnant at time of admission. We chose to use an oxygen saturation of 92% as our cutoff for inclusion. Although severe hypoxemia is less than 90%, the oxygen dissociation curve begins to drop steadily at 92%.^7^ We thus felt most emergency physicians would be uncomfortable discharging a patient with an oxygen saturation of less than 92%.

Along with SARS-CoV-2 testing, standard workup for patients admitted for suspected COVID-19 consisted of a CT of the chest. These scans were either CT angiograms to evaluate for pulmonary embolism, spiral CTs of the chest with intravenous contrast, or a viral chest CT. The viral CTs were low-dose, non-contrast chest CTs with 5-millimeter slices. Based on findings such as multifocal pneumonia, patchy infiltrates, and ground-glass opacities, attending radiologists would classify the viral CTs as category 1 (multifocal pneumonia consistent with SARS-CoV-2), category 2 (indeterminant), or category 3 (not consistent with SARS-CoV-2).

**Figure 1:**
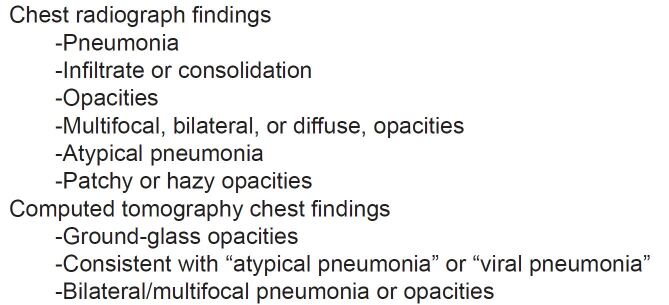
Radiology findings suggestive of SARS-CoV-2 infection.

A priori we defined CXR and CT findings that were known to be associated with SARS-CoV-2 (Figure 1).^8^,^9^ We reviewed the official radiology read for each CXR and CT and categorized the reads by phrases or findings (eg, “ground- glass opacities” or “multifocal pneumonia”). Radiology reads could be classified into more than one group if multiple relevant findings were present. Both CXR and CT were readily available for evaluation of patients with suspected COVID-19 at all EDs in the health system.

Our primary outcome was respiratory decompensation at 72 hours. Respiratory decompensation was defined as the need for supplemental oxygen of any type, high-flow nasal cannula, noninvasive ventilation (bilevel positive pressure or continuous positive pressure), or endotracheal intubation within 72 hours of admission. Only patients who were still in the hospital at 72 hours were included in the primary outcome. Patients could flow across groups during the data analysis. For example, if a patient was on nasal cannula on day one and high-flow nasal cannula on day two, the subject would be listed in their respective group during that time. The same was true of de-escalation of respiratory support.

We performed subgroup analyses for rates of decompensation at 24 and 48 hours as well as for those patients who had a positive SARS-CoV-2 nasopharyngeal PCR test. We also reviewed patients who had a CXR without acute cardiopulmonary findings but an abnormal chest CT consistent with COVID-19 (e.g., ground-glass opacities, multifocal pneumonia, patchy opacities).

## RESULTS

**Figure 2:**
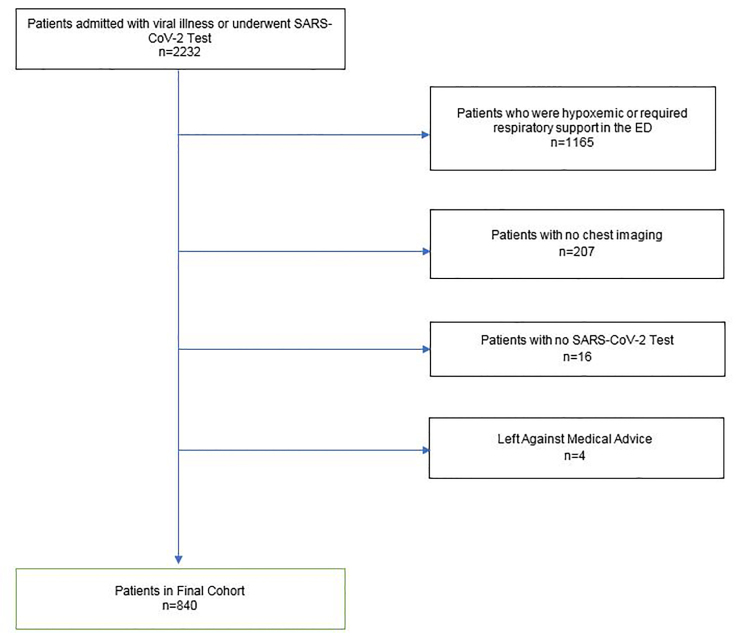
Enrollment diagram.

Between March 1–May 5, 2020, 2232 patients were admitted to TUHS with suspected COVID-19. Of those patients, 840 met our inclusion criteria (Figure 2); 392 (46%) were female and 247 (29%) were over 65 years of age (Table 1). Of these patients, 376 (45%) tested positive for SARS-CoV-2. Of the 840 admitted patients with suspected COVID-19 who were not hypoxemic in the ED, 410 were still admitted to the hospital at 72 hours. Sixty (7%) patients met our outcome for decompensation (Table 3). In the confirmed SARS-CoV-2-positive group, 3% of patients required respiratory support at 72 hours. Table 4 lists the various respiratory inventions in the SARS-CoV-2 positive and negative groups at each time point. At 48 hours, 98 (11.2%) of admitted patients with suspected COVID-19 required oxygen therapy with 57% given nasal cannula and the other 43% on a higher level of respiratory support. At 24 hours, 43 patients (5.1%) needed respiratory support with high-flow nasal cannula, non-rebreather oxygen mask, noninvasive ventilation, or mechanical ventilation. At the time of data analysis, 97 patients were still in the hospital, 49 patients in the SARS-CoV-2 positive group and 48 in the SARS-CoV-2 negative group.

Nine patients (0.8%) in our cohort died during their hospital admission. Seven patients were SARS-Cov-2 positive. The characteristics of these seven patients are listed in Table 6. Six of the seven patients had significant comorbidities including severe congestive heart failure, diabetes, and end-stage renal disease. Only one patient had no apparent comorbidities that would have contributed to death from COVID-19. Of the seven patients who were SARS-CoV-2 PCR positive, four had initial CXRs that were read as no acute cardiopulmonary disease by the attending radiologist. All seven patients had abnormal chest CTs.

A total of 154 patients (18.9%) had normal CXRs in the ED (Table 5); 54 patients of these patients tested positive for SARS-CoV-2 and 18 decompensated at 72 hours. Of the 18 SARS-CoV-2 positive patients who decompensated, 15 had chest CTs, and 13 of those CTs were consistent with SARS-CoV-2. Only one patient with a normal CT of the chest decompensated by 72 hours.

## DISCUSSION

Our hospital system provides a unique insight into the clinical course of COVID-19 as we admitted patients that many other hospitals discharged directly from the ED. In consultation with our pulmonary department, which supervised the care of all COVID-19 patients admitted to the hospital, we chose to admit any patients with abnormal chest imaging as it was felt these patients were at high risk of decompensation, including those who were normoxic in the ED. We also admitted all patients with febrile respiratory rates who were undomiciled or lived in a congregate setting because the public health infrastructure in Philadelphia lacked resources to isolate these patients outside of the hospital. We only tested patients for SARS-CoV-2 who were being admitted to the hospital.

Nearly 1% of patients in this seemingly low-risk cohort died during their hospital admission. This is surprisingly high for patients who were not hypoxemic on admission.^10^ Moreover, this was a fairly young cohort with 70% of patients under 65 years of age. Even more concerning was the fact that four of the seven SARS-CoV-2 positive patients who died had normal initial CXRs. This would suggest that chest radiograph is not nearly sensitive enough to screen for COVID-19 in the normoxic patients. All seven of the COVID-19 patients who died had abnormal chest CTs, which suggests that CT may be a superior modality for screening for COVID-19 disease. This finding is consistent with other published research.^11^

Less than half of the patients who were admitted to the hospital with suspected COVID-19 tested positive for the virus. We believe the low sensitivity of the PCR nasopharyngeal swab for SARS-CoV-2 explains why there was essentially the same number of patients who decompensated in the SARS-CoV-2 positive and SARS-CoV-2 negative groups.^12^ All of the patients included in this study had a diagnosis of viral pneumonia or SARS-CoV-2-related illness. In our cohort 460 patients had ground glass opacities (GGOs) on chest CT, the most common finding on chest CT in patients with COVID-19.^13^ Of patients with GGOs on chest CT, 42% tested SARS-CoV-2 negative. A recent study showed that in a cohort of patients who were SARS-CoV-2 positive, nearly 30% showed CT findings prior to a positive SARS-CoV-2 PCR test.^14^ It is certainly plausible that many of these patients would have a positive SARS-CoV-2 test on repeat testing.

Our findings suggest that patients with suspected COVID-19 who are normoxic in the ED but have abnormal imaging, especially abnormal CT imaging, should be admitted for observation and further care. Seven percent of patients who were normoxic in the ED required respiratory support at 72 hours. The next step in research would be to develop a tool to identify which normoxic patients will decompensate and require oxygen support within 72 hours. Burdick et al developed a machine-learning algorithm that combined 12 variables to predict which patients admitted with COVID-19 would require mechanical ventilation.^15^ Haimovich et al published the Quick COVID-19 Severity Index, a simple three- step scoring model to predict respiratory decompensation at 24 hours.^16^ This index showed moderate sensitivity but allowed patients who were on oxygen by nasal cannula to still receive a low severity score. Most EDs, however, do not have the resources to discharge hypoxic patients requiring oxygen. While these predictive models are applicable to the ED setting, more work remains to be done to capture all patients who may decompensate.

Our cohort does not represent the full spectrum of COVID-19 disease presenting to our ED as many patients were discharged directly from the ED or from an ED screening tent. The screening tent, which housed a physician or advanced practice provider and a nurse, was open during select times based on available staffing. Low-acuity patients with respiratory symptoms were identified upon arrival to the ED and directed to the tent. Patients evaluated in the tent were either discharged directly from the tent or directed back into the ED for further evaluation and treatment at the discretion of the screening provider. Furthermore, no imaging was mandated for patients with COVID-19 who were not being admitted to the hospital. Some clinicians likely ordered more CXRs and viral CTs than other clinicians. While some patients who underwent chest imaging may have appeared sicker to the treating clinician, we attempted to normalize this by looking at only normoxic patients.

At times, hospitals will reach capacity in their ability to care for patients with COVID-19, as resources such as inpatient care space and staff are finite but demand from patients is not. In the first wave of COVID-19, overall ED volume was down at our hospital, allowing us to increase the depth of workup for patients with3/6/2 suspected SARS-CoV-2 infection. In a second wave of COVID-19, we may be forced to more judiciously triage our limited ED and hospital resources. While discharging patients with suspected COVID-19 based on a normal oxygen saturation and normal CXR may not be an optimal strategy, it must be considered. The question of admission vs discharge of the normoxic patient must be based not only on the constraints of a healthcare system during a pandemic, but also the patient’s comorbidities and the ability of a patient to self-monitor symptoms at home. The use of home pulse oximetry may be a viable way to monitor clinical status in a non-clinical setting and provide early identification of a group at risk for respiratory decompensation.^17^

## LIMITATIONS

This study has several limitations to consider. We did not include patients who were triaged away from the ED in our screening tent or who were discharged from the ED without imaging. Radiology reads are highly variable and there is often moderate inter-rater reliability, especially related to CT imaging in COVID-19.^18^,^19^ We relied on these reads to categorize chest CT and CXR findings. This could have affected the internal validity of our study. Our protocol of performing screening chest CTs on all admitted patients may certainly not be applicable to other hospitals.

Further, only patients in the hospital at 24, 48, and 72 hours were included in the analysis. It is possible that a patient discharged before 72 hours decompensated and either went to a different ED or died at home. In addition, 97 patients were still in the hospital at the time of data analysis, so it is possible that these patients died later. We were unable to determine why a patient was placed on a mode of ventilation once admitted to the hospital. Hospital physicians may have differing thresholds for administering oxygen by nasal cannula or other means. We did not control for this factor, and it could have affected the internal validity of our research.

Because our admission order set included a default order for nasal cannula, we were unable to differentiate which patients actually required nasal cannula at 24 hours and which patients simply had an order for nasal cannula. Therefore, data for nasal cannula at 24 hours was not reported. After 24 hours, nasal cannula use was routinely recorded in the electronic health record by the floor nurses and respiratory therapists. In addition, our overall patient volume was down during the initial wave of the pandemic. Finally, our research was conducted in a three-hospital urban health system. Thus, our conclusions may not be externally valid in other geographic settings or in hospitals that do not have the same resources available.

## CONCLUSION

In our data set of suspected patients with COVID-19 who were not hypoxemic on admission to the hospital, 7.1% who remained hospitalized at 72 hours required respiratory support. Many of the patients who decompensated had normal chest radiographs on admission. Further analysis needs to be done on the risk factors that could identify those patients at risk for decompensation vs those patients who could be safely discharged from the ED.

## Figures and Tables

**Table 1:** Demographics [n (%)] of patients tested for severe acute respiratory syndrome coronavirus 2.

	All patients	SARS-CoV-2 positive (n = 376)	SARS-CoV-2 negative (n = 464)
Male	450 (53.6)	193 (51.3)	257 (55.4)
Female	390 (46.4)	183 (48.7)	207 (44.6)
Mean age	56.6 years	55.2 years	57.8 years
Comorbidities			
DM	282 (33.6)	130 (34.6)	152 (32.8)
COPD	78 (9.3)	24 (6.4)	54 (11.6)
Age > 65	247 (29.4)	107 (28.5)	144 (31.0)
BMI 25–30	234 (27.9)	110 (29.3)	123 (26.5)
BMI 30–35	174 (20.7)	92 (24.5)	82 (17.7)
BMI >35	168 (20.0)	90 (24.0)	76 (16.4)
LOS			
Mean (hours)	126 hours	136 hours	117 hours
<24	100 (11.9)	17 (4.5)	83 (17.9)
24–48	186 (22.1)	67 (17.8)	119 (25.7)
48–72	144 (17.1)	80 (21.3)	64 (13.8)
>72	410 (48.8)	211 (56.1)	199 (42.9)

*DM*, diabetes mellitus; *COPD*, chronic obstructive pulmonary disease; *BMI*, body mass index; *LOS*, length of stay.

**Table 2:** Radiology findings.

	All patients	SARS-CoV-2 PCR positive	SARS-CoV-2 PCR negative
Chest radiograph			
Pneumonia	102 (12.1)	67 (17.8)	35 (7.5)
Infiltrate/consolidation	75 (8.9)	36 (9.6)	39 (8.4)
Opacities	241 (28.7)	134 (35.6)	107 (23.1)
Multifocal/bilateral/diffuse	173 (20.6)	110 (29.3)	63 (13.6)
Atypical PNA	27 (3.2)	21 (5.6)	6 (1.3)
Patchy/hazy/ill defined	151 (18.0)	96 (25.5)	55 (11.9)
No acute disease	159 (18.9)	54 (14.4)	105 (22.6)
Chest computed tomography			
Ground-glass opacities	460 (54.8)	265 (70.5)	195 (42.0)
Viral/atypical PNA	331 (39.4)	222 (59.0)	109 (23.5)
Bilateral/multifocal opacities	262 (31.2)	181 (48.1)	81 (17.5)

*SARS-CoV-2*, severe acute respiratory syndrome coronavirus-2; *PNA*, pneumonia.

**Table 3:** Rates of decompensation, n (%).

	At 72 hours	At 48 hours	At 24 hours
Number of patients admitted	410(48.8)	554(66.0)	740(88)
SARS-CoV-2 positive	212	292	358
SARS-CoV-2 negative	198	262	352
Number of patients requiring respiratory support			
SARS-CoV-2 positive	27(3.3)	47(5.6)	-
SARS-CoV-2 negative	33(416.6)	51(6.1)	-

*SARS-CoV-2*, severe acute respiratory syndrome coronavirus-2.

**Table 4:** Respiratory interventions.

Time frame	Respiratory intervention														

	NC			HFNC			NRB			NIV			Vent		
				
	All	+	−	All	+	−	All	+	−	All	+	−	All	+	−
At 24 hours	--	--	--	21 (1.8)	9 (1.9)	12 (1.7)	35 (3.0)	17 (3.5)	19 (2.8)	38 (3.3)	11 (2.3)	27 (3.9)	51 (4.4)	7 (1.5)	44 (6.4)
At 48 hours	45 (3.9)	26 (5.4)	19 (2.8)	12 (1.0)	5 (1.0)	7 (1.0)	15 (1.3)	7 (1.5)	8 (1.2)	10 (0.9)	3 (1.5)	7 (1.2)	16 (1.4)	6 (0.6)	10 (1.5)
At 72 hours	26 (2.2)	15 (3.1)	11 (1.6)	8 (0.7)	3 (0.6)	5 (0.7)	11 (0.9)	6 (1.2)	5 (0.7)	8 (0.7)	2 (0.4)	6 (0.9)	7 (0.6)	1 (0.2)	6 (0.9)

Respiratory interventions within the first 24, 48, 72 hours, divided by type of intervention and COVID-19 test result. (+) = COVID test positive; (−) = COVID-19 test negative; All = Includes COVID-19 positives and negatives; Values reported as # (%). Adjusted for length of stay.

*NC*, nasal cannula; *HFNC*, high-flow nasal cannula; *NRB*, non rebreather mask; *NIV*, noninvasive ventilation; *Vent*, ventilator

**Table 5:** COVID-19 patients with normal chest radiographs (n = 54).

	n	Decompensation	No Decompensation
Chest CT consistent with SARS-Cov-2	39	13 (33%)	26
Chest CT not consistent with SARS-Cov-2	4	1 (25%)	3
Indeterminate chest CT	7	1 (14%)	6
No chest CT	4	1 (25%)	3

*SARS-CoV-2*, severe acute respiratory syndrome coronavirus 2; *CT*, computed tomography.

**Table 6:** SARS-CoV-2 positive mortality group.

Age	Gender	Major comorbidity	Intubated on	Died	Initial CXR	Initial chest CT
78	Male	Severe CHF (EF=20%)	Day 15	Day 15	Abnormal	Abnormal
67	Female	None	Day 5	Day 21	Abnormal	Abnormal
51	Male	Diabetes	Day 3	Day 31	Normal	Abnormal
46	Female	ESRD	Day 7	Day 19	Normal	Abnormal
81	Male	Hepatocellular carcinoma	Comfort care	Day 11	Normal	Abnormal
68	Male	Diabetes (DKA)	Day 7	Day 8	Normal	Abnormal
84	Female	Post-polio paralysis	Comfort care	Day 7	Abnormal	Abnormal

*CXR*, chest radiograph; *CT*, computed tomography; *CHF*, congestive heart failure; *EF*, ejection fraction; *ESRD*, end-stage renal disease; *DKA*, diabetic ketoacidosis.
